# Privacy-Aware Synthetic Tabular Data Generation for Healthcare: Application to Sepsis Detection

**DOI:** 10.3390/bioengineering13050511

**Published:** 2026-04-28

**Authors:** Eric Macias-Fassio, Aythami Morales, Cristina Pruenza, Julian Fierrez, Carlos Espósito

**Affiliations:** 1 Instituto de Ingeniería del Conocimiento, 28049 Madrid, Spain; cristina.pruenza@iic.uam.es; 2 BiometricsAI, Universidad Autonoma de Madrid, 28049 Madrid, Spain; aythami.morales@uam.es (A.M.); julian.fierrez@uam.es (J.F.); 3 Department of Mathematics, Universidad de las Palmas de Gran Canaria, 35018 Las Palmas de Gran Canaria, Spain; 4 Universidad Autonoma de Madrid, 28049 Madrid, Spain; carlos.esposito@uam.es

**Keywords:** synthetic data, machine learning, sepsis detection

## Abstract

Background: Machine learning-based Artificial Intelligence (AI) models have shown significant potential in the biomedical field, offering promising advances in diagnostics, personalized medicine, and patient care. However, to build these models, we have to deal with important challenges, including (1) the scarcity and low quality of available datasets in many important applications and (2) privacy concerns associated with sensitive patient data. Synthetic data (SD) generation has emerged as a promising strategy to address these challenges, yet many existing approaches struggle to simultaneously preserve privacy and accurately model tabular data, the predominant format in healthcare. Methods: We propose Kernel Density Estimation–K-Nearest Neighbors (KDE-KNN), a privacy-aware tabular data generation method, and evaluate its performance against state-of-the-art techniques. Using sepsis detection as a real-world case study, we assess both data utility and privacy protection. Results: Models trained on KDE-KNN-generated SD outperformed those trained on real data across both internal testing and external validation. In particular, a support vector machine achieved superior performance when trained on SD relative to real data. This gain is likely driven by the balanced class distribution of the synthetic dataset, underscoring KDE-KNN’s utility as an effective data balancing strategy. Consistent performance in external validation further supports the robustness and generalizability of the proposed approach. Privacy evaluation indicated a lower re-identification risk, with a mean distance to closest record of 4.971 between synthetic and real samples, compared with 2.715 among real samples. Conclusions: KDE-KNN effectively captures underlying population distributions while generating high-quality SD that preserve statistical fidelity and protect sensitive information. By balancing the trade-off between utility and privacy, the method produces representative datasets without exposing individual records. These findings position KDE-KNN as a valuable tool for data-scarce and privacy-sensitive applications, with broad potential across healthcare and other data-driven domains.

## 1. Introduction

The U.S. Food and Drug Administration (FDA) approved 1016 Artificial Intelligence (AI)/Machine Learning (ML) Enabled Medical devices [1] between 1995 and 2024 (https://www.fda.gov/medical-devices/software-medical-device-samd/artificial-intelligence-and-machine-learning-aiml-enabled-medical-devices (accessed on 15 November 2025)), with a significant increase in the last few years. This trend highlights the potential of AI models as a promising technology in the healthcare sector [2,3]. The ability of AI to process large volumes of data efficiently and accurately has changed medical research, improved early diagnostics, and improved treatments [4,5]. As a result, AI-driven approaches have shown remarkable performance in the detection of a wide variety of diseases, including cancer [6,7], cardiovascular disease [8], Parkinson’s disease [9,10], liver cirrhosis [11], and other conditions such as fatigue [12]. Since Google’s paper Attention Is All You Need was published [13], the field has undergone a paradigm shift toward generative AI and foundation models. One of the advantages of foundation models is the ability to perform tasks for which they were not explicitly trained, demonstrating strong generalizability. However, the development of predictive or generative AI models in the medical domain faces significant challenges. One of the primary obstacles is the difficulty in accessing large-scale and diverse datasets [14].

In addition, many governments are introducing strict regulations for personal data processing and AI applications such as the new AI Act of the European Union (https://artificialintelligenceact.eu/ (accessed on 20 November 2025)), CCPA (https://oag.ca.gov/privacy/ccpa (accessed on 21 November 2025)) (Unitated States) and LGPD (https://www.gov.br/anpd/pt-br/centrais-de-conteudo/brazilian-data-protection-law.pdf (accessed on 28 November 2025)) (Brazil), which enforce data protection measures. A significant development in the regulatory landscape of AI has occurred with the enactment of the AI Act within the European Union. This legislative framework is designed to oversee and govern the application of AI models. In the domain of biomedical research, caution must be exercised when employing patient data for the training of AI models. Patient data, characterized by its sensitive nature [15,16], is subject to strict protection under data protection laws, requiring the preservation of privacy [17,18].

Related research in privacy-aware and privacy-preserving methods can be found in the literature on biometric person recognition [18,19,20], and we can also see increasing efforts in dealing with privacy elements in large AI models, including visual models [21], Large Language Models (LLMs) [22,23], and Vision Language Models [24]. In addition, recent work such as Robust Health [25] has proposed non-interactive privacy-preserving systems for heterogeneous mobile health diagnosis, highlighting the feasibility of secure and scalable AI deployment in clinical environments.

A solution that can potentially overcome these limitations involves the generation of fully SD as an alternative to real data [26]. SD are artificial data generated by a trained model and built to replicate real data taking into account its distribution (mean, variance) and structure (e.g., correlation between attributes) [27]. The use of SD generation emerges as a versatile methodology in AI, allowing the augmentation of datasets to improve model training [28,29] and safeguarding the privacy of sensitive information [30]. Indeed, Gartner predicts the dominance of SD over real data in AI models by 2030 (https://www.gartner.com/en/newsroom/press-releases/2022-06-22-is-synthetic-data-the-future-of-ai (accessed on 10 December 2025)).

**Figure 1:**
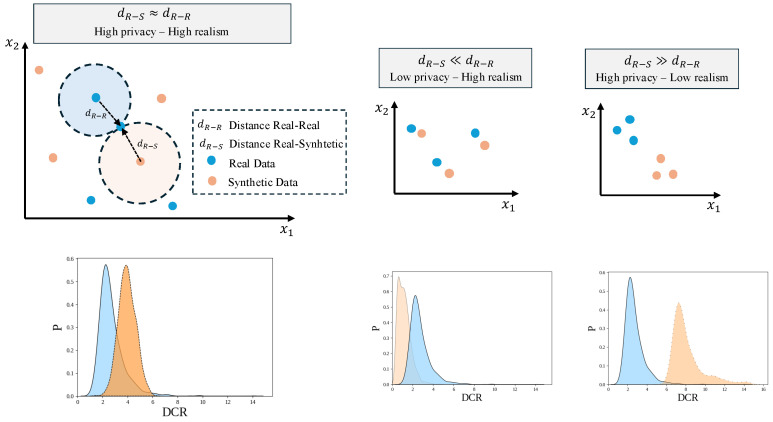
Compromise between privacy and realism of synthetic samples. The graphs illustrate the distance between real and synthetic samples in a conceptual 2-dimensional space. The top row shows different regimes based on the relationship between real–real and real–synthetic distances. The bottom row presents the corresponding distributions of the Distance to Closest Record (DCR), where blue represents real data and orange represents synthetic data. P denotes the probability density, providing a quantitative interpretation of how shifts in distance reflect changes in similarity between real and synthetic data.

Privacy-preserving methods for generating synthetic tabular data are of critical importance in biomedical research, where data sensitivity and regulatory constraints often limit data sharing and reuse [31,32]. A central challenge in privacy-preserving SD generation is achieving an optimal trade-off between data utility and privacy. High levels of privacy protection, such as those enforced through strict differential privacy parameters, can significantly degrade the statistical fidelity and predictive value of synthetic datasets, limiting their usefulness for downstream biomedical analyzes. In contrast, prioritizing utility without adequate privacy safeguards can lead to the leakage of sensitive patient information. In biomedical applications, where both the integrity of data and the protection of individual privacy are paramount, careful balance of this trade-off is essential. The goal is to generate SD that are sufficiently representative to support meaningful research and clinical insights, while ensuring that the risk of re-identification or inference of personal information remains acceptably low. Figure 1 illustrates the compromise between privacy-preserving generation and the realism of synthetic samples.

This work extends our previous study [31], where the Kernel Density Estimation–K-Nearest Neighbor (KDE-KNN) method was initially introduced. In the present paper, we provide a more comprehensive evaluation, including enhanced privacy mechanisms, comparison with additional state-of-the-art methods, and validation on an external dataset. In more detail, the main contributions of this work are the following:

- We improved and analyzed the performance of the KDE-KNN SD generation method to train and evaluate supervised learning algorithms.
- We extensively evaluated state-of-the-art SD generation methods in terms of utility and privacy in the context of sepsis detection. Our results show that the privacy-aware method KDE-KNN, outperforms existing approaches in generating synthetic tabular data for sepsis detection.
- Using two real databases with more than 2000 patients, we evaluated the generalizability of SD generation methods. Our results suggest that KDE-KNN has certain advantages in terms of generalization over other methods.

The remainder of the paper is organized as follows. Section 2 reviews previous research on synthetic tabular data generation methods and ML models for the prediction of sepsis. Section 3 describes the databases, supervised ML models, and data generation approaches used in this study. Section 4 details the experimental protocol and presents the results. Section 5 provides a discussion of the findings, including limitations and future research directions. Finally, Section 6 summarizes the main conclusions of the work.

## 2. Related Works

We have divided this section into two parts: (i) Synthetic Tabular Data generation approaches in Healthcare, and (ii) ML models for predicting sepsis.

### 2.1. Synthetic Tabular Data Generation Approaches in Healthcare

In 1993, Rubin [33] and Little [34] proposed a statistical method to generate synthetic value records instead of using real data to avoid privacy concerns. Today, the concept of SD has evolved to include artificial data generated by trained models. This SD is designed to replicate real-world datasets by accurately capturing their distributional properties (such as mean and variance) as well as their structural characteristics (such as correlations between different attributes) [27]. SD generation stands out as a highly promising yet largely underexploited technology for fulfilling privacy-preserving laws or augmenting datasets to enhance model performance.

The present study focuses on the generation of synthetic tabular data from electronic health records (EHR). The tabular healthcare-related data stored in the EHR contain vast and diverse amounts of patient data. Typically, each row in a healthcare tabular dataset represents a single data record that contains descriptive patient details such as date of birth, gender, and demographic information, along with sensitive attributes primarily consisting of longitudinal data. These longitudinal data comprise a series of medical events that occur at various time points, encompassing diagnoses, laboratory test results, and prescription information [35]. Tabular data are inherently heterogeneous, often comprising continuous, discrete, and categorical features that each adhere to distinct statistical distributions. This complexity introduces significant challenges in the accurate synthesis of such data.

In the healthcare context, numerous approaches to generating SD can be found in the literature. Among these, one widely utilized algorithm is the Synthetic Minority Oversampling Technique (SMOTE). This algorithm operates by synthesizing new data by interpolating existing samples. An extension of this algorithm is SMOTE–TOMEK [36], which incorporates Tomek Links, an under-sampling technique to remove synthetic noise samples. Another statistical approach to generate SD involves KDE based models. Our framework for SD generation in the healthcare context is based on KDE, chosen for its non-parametric nature and demonstrated efficacy, particularly in small datasets, which are prevalent in the biomedical field [37].

Additional methodologies used for SD generation involve generative models, which include Generative Adversarial Networks (GANs), Diffusion Models (DMs), and LLMs.

Since their inception in 2014 [38], GANs have demonstrated exceptional capability in the production of synthetic image data [39]. For this reason, the application of GANs to other data types, such as tabular data, is a popular topic in the AI research community [40]. One of the most widely used GAN-based synthetic tabular data generation approaches is CT-GAN, a Conditional Tabular Generative Adversarial Network [41], which introduces a mode-specific normalization to handle columns with complicated distributions.

On the other hand, DMs represent another class of generative models which have been widely used in the computer vision field. In particular, recent advances have led to the development of architectures tailored to exploit DMs for tabular data, such as TabDDPM [42], which has demonstrated significant potential and promising results in this regard. Furthermore, with the emergence of LLMs, capable of performing tasks beyond their explicit training, we have analyzed whether models such as GPT-4 can generate accurate SD derived from the information on which they were trained. Although the potential for SD is huge, there are important considerations regarding the quality, reliability, and ethical implications of using SD. Ensuring that the generated data are representative, unbiased, and compliant with relevant legal and ethical standards is a critical challenge. For these reasons, in this study we have evaluated the quality and privacy consideration of SD generated by generative models based on SMOTE–TOMEK, TabDDPM, CT-GAN, CHAT-GPT, and KDE.

### 2.2. Machine Learning Models for Sepsis Prediction

Sepsis is defined as a life-threatening organ dysfunction caused by a dysregulated host response to infection [43]. Each year, sepsis is responsible for an estimated 11 million deaths (https://www.who.int/news-room/events/detail/2024/09/13/default-calendar/launch-of-2030-global-agenda-for-sepsis-and-world-sepsis-day-2024 (accessed on 20 January 2026)). The importance of detecting sepsis early is crucial, as it is a time-sensitive clinical process. There is a direct relationship between early diagnosis and improved results, measured by lower mortality, better patient prognosis, and reduced use of hospital resources [44], but early and accurate detection of sepsis is still a challenging clinical problem [45]. For this reason, several ML algorithms have been designed to predict sepsis using retrospective data [46,47,48,49,50,51]. In addition, clinically interpretable approaches such as the LIP score [52] have been proposed as screening tools based on biomarkers including lymphocyte count, international normalized ratio and procalcitonin, highlighting the importance of combining predictive performance with clinical interpretability.

In our study, we try to predict whether a patient will have or not sepsis. We frame the task of sepsis detection as a classification problem, with the aim of addressing the question: Will patient A develop sepsis in the future? We substantiate our findings through validation in an external cohort for robustness and generalization.

## 3. Materials and Methods

In this study, we have used 2 databases: (i) the Mannheim database (MaDB) used for training our models and building synthetic datasets; (ii) the Son Llàtzer hospital database (SLDB) used as an external validation dataset to evaluate the generalization capacity of the trained models.

### 3.1. Mannheim Database

We used the University Medical Centre Mannheim database (MaDB) of patients admitted to Intensive Care Unit (ICU) [53]. This database contains a total of 1275 patients, 979 with non-sepsis and 296 with sepsis. Initially, the MaDB comprised 42 timelines of features and the diagnosis of sepsis at each time step. However, for comparative analysis with the SLDB, it was necessary to align the feature sets. Consequently, only 27 features were found to be common between both databases. These features include both patient demographics (e.g., age, sex) and laboratory results (Table 1).

The MaDB contains temporal data that allow a precise tracking of the times of onset of sepsis in patients, as evidenced in Table 2. The notable variability in the timing of sepsis manifestation within this dataset underscores its inherent heterogeneity. However, we do not use this temporal information, because we treat the detection of sepsis as a binary classification problem. Instead, we set a cut-off value at 9 h as we estimated that in this time period all clinical tests could be performed and laboratory results could be collected. If a test has been performed several times during this period, the last value is used. In this way, we constructed a dataset in which our predictor variables were collected in that time interval and the objective was to predict whether or not a patient will develop sepsis in the future (classification problem). The MaDB has been used to train and test models and generate SD.

### 3.2. Son Llátzer Hospital Database

We used a database from Son Llàtzer Hospital of patients admitted to the emergency department and the ICU. The Son Llàtzer database (SLDB) contains 2028 patients in total, 1014 with non-sepsis and 1014 with sepsis. In this database, we also selected the 27 common features with MaDB. However, within the SLDB, the precise mean time to the onset of sepsis remains unknown. According to insights from the medical team, the mean time to onset of sepsis is estimated to range between 24 and 48 h. We used this database for external validation, acknowledging significant disparities in the onset times of sepsis compared to our primary dataset. In particular, there are substantial variations in the data distribution between the two databases. Thus, we perceived this as an opportunity to assess the generalizability of our models across diverse demographic populations.

### 3.3. Dataset Shift

To quantify distributional differences between cohorts and assess dataset shift, we performed the Mann–Whitney U test for each feature, as it is a non-parametric test suitable for comparing variables with potentially non-Gaussian distributions. To account for multiple comparisons across the 27 variables, *p*-values were adjusted using the Bonferroni correction.

### 3.4. Data Preprocessing

Prior to model training, a preprocessing pipeline was applied to ensure data quality and consistency across both datasets. Missing values were imputed using the median of each feature, as this approach is robust to skewed distributions and reduces the influence of extreme values. Subsequently, all variables were standardized using z-score normalization to ensure comparability across features and to improve the stability and performance of the ML models.

### 3.5. Sepsis Prediction Models

Early detection of sepsis is critical as it is a time-sensitive clinical process. There is a direct relationship between early identification and better patient outcomes. However, traditional methods have significant limitations for the early detection of sepsis. Consequently, the integration of AI and ML techniques capable of processing large volumes of data efficiently and accurately is transforming early medical diagnosis. Our study evaluates three distinct ML models and assesses their performance based on the Area Under the Curve (AUC) score.

- Random Forest (RF). It is a widely used ML algorithm that belongs to the ensemble learning family, characterized by the construction of multiple decision trees during training. For classification tasks, the RF outputs the predicted class, which in the context of sepsis prediction means whether a patient is likely to develop sepsis or not.
- Support Vector Machine (SVM). Unlike traditional classifiers that aim to find a decision boundary that separates classes, SVM seeks to find the hyperplane that best divides the classes while maximizing the margin between them. In our experiments, we have used two SVM changing the type of kernel: (i) SVM with a linear kernel; (ii) SVM with a radial basis function (rbf) kernel.
- Gradient Boosting Algorithm. Such as XGboost or Catboost that combines multiple learners, usually decision trees to form an ensemble. Each tree is constructed in a sequence, where subsequent trees focus on the errors made by previous trees.

The hyperparameters of the models were tuned using the Optuna library [61]. Specifically, we used a TPE (Tree-structured Parzen Estimator) sampler with 40 trials to maximize AUC. The hyperparameter grid and the optimal hyperparameters for each model have been included in Appendix A.

To improve the interpretability of the ML models, we employed Shapley Additive Explanations (SHAP), a widely used explainable artificial intelligence (XAI) method [62,63,64]. SHAP values are based on cooperative game theory and quantify the contribution of each feature to the model’s prediction.

### 3.6. Data Modeling Approaches

In this paper, we have analyzed 5 SD generation methods:

SMOTE–TOMEK [65]: SMOTE–TOMEK is a method that combines oversampling and under-sampling techniques to generate synthetic samples. Initially, this method was used to generate observations exclusively from the minority class, but we expanded this approach to incorporate the majority class as well, resulting in the creation of a fully synthetic dataset.

TabDDPM [42]: TabDDPM is a design of probabilistic diffusion denoising models for tabular data. To tackle mixed-type characteristics of tabular data, this architecture integrates gaussian diffusion to capture the characteristics of continuous features and multinomial diffusion to effectively model categorical attributes.

KDE: KDE is a method used to estimate probability density functions. By constructing this distribution, we gain the ability to generate SD samples by sampling. This capability allows for the creation of synthetic datasets representative of the underlying probability distribution. We conducted experiments using multivariate KDE, taking into account the interdependencies between features. This allows us to capture complex relationships and dependencies across multiple variables simultaneously.

CHAT-GPT [66]: LLMs are known for their ability to perform tasks for which they were not explicitly trained. For this reason, we analyzed whether CHAT-GPT would generate truthful synthetic samples. To do so, we used 3 versions of CHAT-GPT (o1, o3-high, and 4o).

CT-GAN [41]: It is a Conditional Tabular Generative Adversarial Network which implements a mode-specific normalization to overcome the non-gaussian and multimodal distribution, one of the main challenges when modeling continuous distributions. In addition, they implement a training-by-sampling method to handle underrepresented categories in the categorical columns.

### 3.7. Synthetic Data Evaluation

We evaluated the SD generated by each method considering both quality and privacy. To evaluate quality, we trained several ML algorithms and analyzed their performance on real data. To evaluate privacy preservation, the Distance to Closest Record (DCR) metric was used. The DCR calculates the average distance between the synthetic samples and their closest real data points.

Additionally, we assessed the fidelity and diversity of the generated data using the density and coverage metrics [67]. Both metrics are computed in a feature space using KNN with k=5. Density measures the fidelity of synthetic samples by quantifying how densely they populate the neighborhoods of real data points in a feature space. Density is not upper bounded by 1. Coverage measures the diversity of the SD by computing the proportion of real samples that are within the neighborhood of at least one synthetic sample. Coverage is bounded between 0 and 1.

### 3.8. KDE-KNN: Privacy-Aware Synthetic Clinical Data

Our proposed methodology is based on the integration of the KDE and the KNN ML algorithm. The core idea is to use a multivariate gaussian KDE to approximate the probability density function of the original dataset features and then sample it to generate synthetic datasets. However, because the feature space can be very large, we trained a KNN to validate the synthetic samples. Let $D_{train}={\left\{x_{i}\right\}}_{i=1}^{N}$ and $D_{test}={\left\{x_{i}\right\}}_{i=1}^{M}$ be the training and test real dataset, where $x_{i}\in R^{d}$. The procedural steps to construct our synthetic dataset are the following:

1. Step 1—Training a KNN model (*M*): A KNN model was trained using the real training dataset $D_{train}$.
2. Step 2—Data preparation for KDE: The training dataset $D_{train}$ was partitioned into two distinct groups, patients with sepsis $D_{train}^{S}$ (18.55% of users) and without sepsis $D_{train}^{C}$ (81.45% of users).
3. Step 3—Multivariate KDE construction ($\hat{p}\left(x\right)$): two statistically independent multivariate KDE distributions (${\hat{p}}^{S}\left(x\right)$) and (${\hat{p}}^{C}\left(x\right)$) were trained for $D_{train}^{S}$ and $D_{train}^{C}$.
  (1) $$\hat{p}\left(x\right)=\frac{1}{Lh^{d}}\sum_{i=1}^{L}K\left(\frac{x-x_{i}}{h}\right),$$
  where K(·) denotes a Gaussian kernel, *h* is the bandwidth parameter controlling the smoothness of the distribution, and *L* is the number of samples. In this study, the bandwidth *h* is selected using Scott’s rule [68].
  (2) $$K\left(u\right)=\frac{1}{{\left(2\pi \right)}^{d/2}}\exp\left(-\frac{1}{2}{∥u∥}^{2}\right)$$
4. Step 4—SD generation: Sampling was performed from each multivariate KDE model, generating 540 synthetic patients with sepsis ($x^{SD-S}$ using ${\hat{p}}^{S}\left(x\right)$) and 540 synthetic patients without sepsis ($x^{SD-C}$ using ${\hat{p}}^{C}\left(x\right)$) and a random seed *x*.
5. Step 5—Utility Validation: Validation of the synthetic samples was conducted for the 1080 synthetic sample (540+540) using the KNN model *M* learned during the Step 1. Any misclassified sample was discarded as a non-usable.
6. Step 6—Privacy Validation (σ parameter): The average DCR for the real dataset was first computed and used as a reference σ.
  (3) $$\mathrm{DCR}\;(\mathrm{real}\;\mathrm{vs}.\;\mathrm{real})=\sigma =\frac{1}{N}\sum_{j=1}^{N}\min_{i\neq j}∥x_{j}-x_{i}∥,\;i,j\in \left\{1,\ldots ,N\right\}$$
  Then, the DCR for each synthetic sample was calculated as:
  (4) $$\mathrm{DCR}\;\left(x_{j}^{\mathrm{SD}-S}\right)=\min_{i\in \{1,\ldots ,N\}}∥x_{j}^{\mathrm{SD}-S}-x_{i}∥,\;j\in \left\{1,\ldots ,540\right\}$$
  The DCR was calculated for all the synthetic samples $\mathrm{DCR}(x_{j}^{SD-S})$ and $\mathrm{DCR}(x_{j}^{SD-C})$. Synthetic samples that were too close to real data (DCR<σ) were subsequently discarded. σ parameter defines the minimum acceptable distance, measured in standard deviations, that a synthetic sample must maintain from the real data, thereby ensuring compliance with the required privacy-aware constraints. The default value is set to 1, which means that the synthetic samples must be at least one standard deviation from any real data point. While the bandwidth parameter controls the smoothness of the estimated probability density, the σ parameter enforces a minimum distance between real and synthetic samples. Together, these parameters regulate the trade-off between distributional fidelity and sample-level similarity. It is important to note that this mechanism provides an empirical privacy control rather than relying on differential privacy frameworks [69]. The DCR constraint acts as a proxy for disclosure risk by limiting the similarity between synthetic and real samples.
7. Step 7—The process (Steps 4–6) was executed iteratively until we reached a total of 540 synthetic usable samples were obtained for both sepsis and without sepsis classes. This process ensures the creation of a balanced synthetic dataset representative of both septic and non-septic patient populations while preserving the utility of the data and reducing the risk of re-identification.

**Figure 2:**
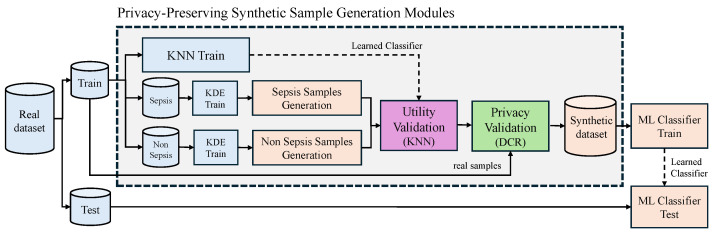
Block diagram of our proposed KDE-KNN method for SD generation including the generation modules based on two Kernel Density Estimators (Sepsis and Non-Sepsis) and KNN sampling.

For clarification, we close this section by visualizing our proposed synthetic method as a flow chart, illustrated in Figure 2.

### 3.9. Computational Complexity

From a computational perspective, different SD generation methods exhibit varying complexity profiles depending on their underlying mechanisms:

- **KDE-KNN:** The cost is driven by density estimation, sample generation, and validation. The iterative sample-generation and validation stage scales approximately as O(TNd), where *N* is the number of real samples, *d* the number of features, and *T* the number of generated candidates. The rejection-based sampling mechanism increases *T* depending on the acceptance rate.
- **SMOTE–Tomek:** The computational cost is dominated by nearest-neighbor searches and scales as $O(N^{2}d+Gd)$ in the naive case, where *N* is the number of samples and *G* the number of generated synthetic instances.
- **TabDDPM:** The generation phase is dominated by the reverse diffusion process and scales as $O(GSC_{\theta })$, where *G* is the number of generated samples, *S* is the number of diffusion steps, and $C_{\theta }$ is the cost of one forward pass of the denoising network. Assuming linear scaling with the feature dimension, this becomes O(GSd).
- **CTGAN:** The generation phase is dominated by the forward pass of the generator network and scales as $O(GC_{G})$, where $C_{G}$ is the cost of a single generator pass. Under linear scaling with *d*, this simplifies to O(Gd).

## 4. Experiments and Results

In this section, we evaluate several SD generation methods and the influence that synthetic samples had on the performance of ML models to detect sepsis.

### 4.1. Experimental Protocol

Initially, our study was based on two distinct sepsis databases: the MaDB and the SLDB. The MaDB served as the primary dataset for model training/testing and SD generation, while the SLDB was used exclusively for external validation purposes.

Our first experimental phase involved evaluating the model performance using real data exclusively. To do this, we used MaDB and partitioned the data into training sets (85%) and testing sets (15%), repeating the experiment three times while changing the seed. Additionally, each partition underwent an external validation using the SLDB. In particular, our analysis revealed that the performance of the ML models remained consistent across different partitions, suggesting a minimal impact of partitioning during model training.

Experiment 2 focused on optimizing the integration of KDE with ML models. Specifically, we evaluated three approaches: KDE-Random Forest (KDE-RF), KDE-Support Vector Machine (KDE-SVM) and KDE-K-Nearest Neighbors (KDE-KNN) in terms of their utility. Among these, KDE-KNN demonstrated the best performance. To conclude this phase of experimentation, we assessed how incorporating real and SD into the training set affected model performance.

Experiment 3 focused on an in-depth analysis of the statistical properties, utility, and privacy of the synthetic samples generated by all methods. We generated a fully synthetic balanced dataset comprising 540 samples with sepsis and 540 samples without sepsis, mirroring the size of our original imbalanced training set. To achieve this, one of the Train-Test partitions from the real data used in Experiment 1 was randomly selected, as that experiment demonstrated stable ML model performance across the three partitions. The training set from the selected partition was then used as the seed for the SD generation methods detailed in Section 3.4. Subsequently, the utility of the SD was assessed by evaluating the performance of the ML models on the reserved test set (real data). The last phase of our experimentation aimed to assess the preservation of the privacy of SD. To do so, we computed the proximity of SD to real data using the Mean Distance to Closest Record (DCR) metric [70]. The mean DCR calculates the average distance between the synthetic samples and their closest real data points.

### 4.2. Experiment 1: Real Data Results (Baseline)

The set of 27 predictive features, along with the statistical differences in their distributions between cohorts, is summarized in Table 2. The results of the Mann–Whitney U test, adjusted using the Bonferroni correction, indicate that the majority of variables exhibit statistically significant differences between the MaDB and SLDB cohorts (*p*-value <0.01). Only a small number of variables, such as creatinine and age, do not show significant differences, indicating a notable distribution shift between the datasets. Table 3 presents the results of Experiment 1, which evaluated the performance of various ML models using only real data in terms of AUC. Among the algorithms evaluated, the Random Forest (RF) model achieved the best overall performance, with a mean AUC of 0.6708±0.0169 on MaDB, 0.6469±0.0313 on SLDB reflecting consistent predictive performance across datasets. The SVM with an rbf kernel showed competitive performance, particularly in SLDB (0.6952±0.0282), slightly outperforming RF in that dataset, suggesting good generalization capabilities in this context. In contrast, the SVM with a linear kernel demonstrated lower performance across both datasets, indicating limited capacity to model non-linear relationships between features. XGBoost performed well on MaDB (0.6547±0.0296), although its performance decreased on SLDB (0.5753±0.0399), possibly reflecting a degree of overfitting or reduced generalizability. Finally, CatBoost achieved the lowest performance among the models tested, with AUC scores of 0.5571±0.053 and 0.5657±0.0349 on MaDB and SLDB, respectively.

**Figure 3:**
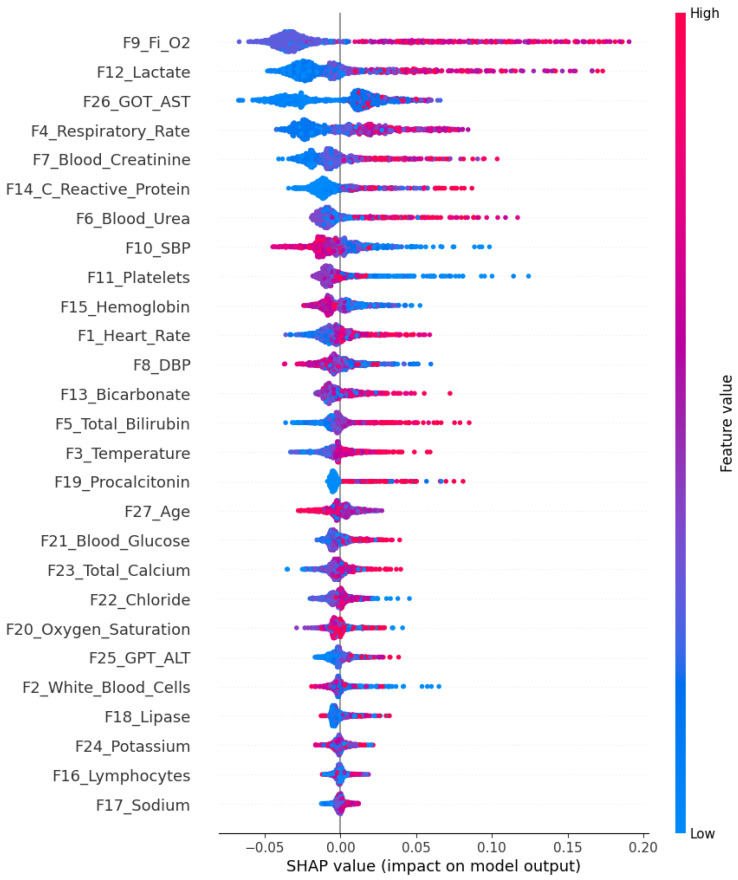
Shap summary plot illustrating the impact of each feature on the RF model output.

Additionally, SHAP analysis (Figure 3) was conducted to include the interpretability of the predictions of the RF model, revealing that features such as F9_Fi_O2, F12_Lactate, and F14_C_reactive_Protein are among the most influential in sepsis detection, a finding that aligns with the existing literature [44,71,72].

### 4.3. Experiment 2: Integrating Synthetic Data with ML Models (Utility)

In Experiment 2, we analyzed the integration of the KDE generation method with 3 supervised classification models evaluating the utility of the SD generated (Table 4). The purpose of the ML model was to validate the quality of the synthetic samples, discarding those of lower quality. To ensure a comprehensive evaluation, we selected ML models from different families: Random Forest (RF), Support Vector Machine (SVM), and KNN; analyzing them in three configurations: KDE-RF, KDE-SVM, and KDE-KNN. From each configuration, we generated SD, trained 5 ML models, and tested these ML models with real data, analyzing their performance in terms of AUC. Across all generation methods, the integration of KDE with downstream ML models led to performance improvements compared to baseline models without augmentation. Among generation strategies, KDE-KNN (Figure 2) achieved the highest overall AUC scores, with the best results observed for SVM (linear) and SVM (rbf) classifiers (0.7093±0.0063 and 0.7128±0.0062 on MaDB, and 0.7540±0.0040 and 0.7632±0.0016 on SLDB, respectively). This shows that even a relatively simple algorithm like KNN can effectively eliminate noisy synthetic samples.

In addition, we wanted to analyze how the combination of real and SD during training affects model performance. We selected the best configuration, KDE-KNN, and trained an SVM model (the algorithm that demonstrated the highest generalization capabilities in Experiment 1) using varying proportions of real and SD. The experiment was carried out 3 times using different seeds and the findings are presented in Table 5. The findings indicate that increasing the percentage of SD in the training set improved the performance of the model, with AUC values increasing from 0.6194 to 0.7160 in MaDB and from 0.6952 to 0.7682 in SLDB. This shows that balancing the training set using SD generated with KDE-KNN enhances the performance of the model.

### 4.4. Experiment 3: Ablation Study

**Figure 4:**
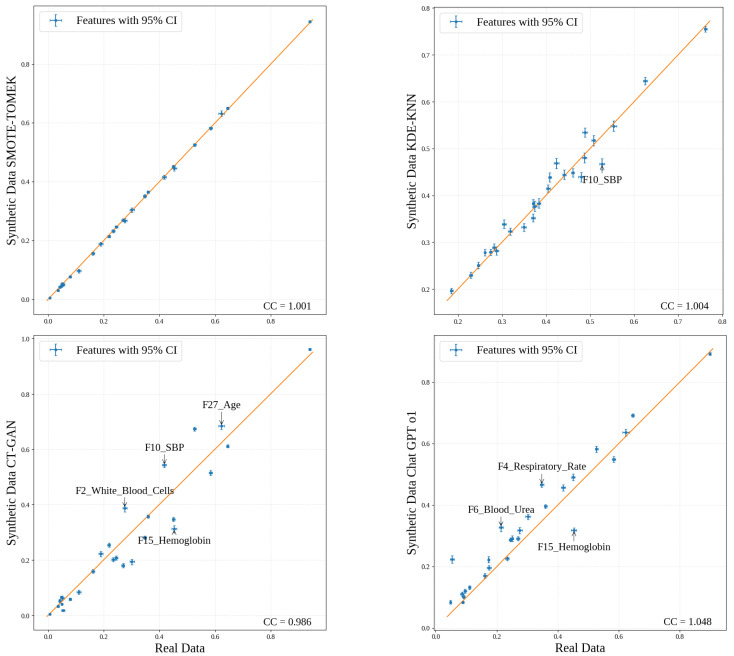
Dimensional means (95% confidence intervals) between synthetic and real data for different synthetic generation models using the MaDB. The orange diagonal line represents the line of perfect agreement between synthetic and real data distributions.

In this section, we conduct an in-depth evaluation of the KDE-KNN method and compare its performance with the SD generation methods mentioned in Section 3.4. First, we compared the dimensions-wise means and variances between the synthetic and real distributions generated by each synthetic method (Figure 4). The results showed that KDE-KNN captured the complexity of the univariant distributions, only outperformed by SMOTE–TOMEK as it is an interpolation method. As we will see in further experiments, privacy is compromised when methods such as SMOTE–TOMEK are used because the statistical distributions of the real and SD remain too close. Additionally, we performed pairwise Kolmogorov–Smirnov tests to determine whether synthetic one-dimensional distributions differ significantly from the real ones. The statistical results demonstrated that all synthetic methods learned univariate distributions that do not differ significantly from the real ones (Table 6).

To further assess the impact of SD on model generalization, we trained multiple ML classifiers using balanced synthetic datasets and evaluated their performance on real-world test data. A detailed comparison of these approaches is provided in Appendix B, where the results are reported for all evaluated methods under a consistent experimental setup, analogous to Experiments 1 and 2. The results indicate that the use of SD significantly improved the discriminative performance of most classifiers, as reflected by higher AUC values compared to those obtained with real unbalanced data. Among the evaluated data generation methods, KDE-KNN consistently achieved the best performance on both datasets. In particular, the SVM with an rbf kernel attained the highest AUC (0.7128 on MaDB and 0.7682 on SLDB), demonstrating the robustness of this method for generating high-quality synthetic samples that preserve the statistical properties of the original data distribution while effectively balancing class representation. In contrast, the SD produced by the CHAT-GPT-based models (o1, o3-high, and 4o) exhibited poor utility for model training, leading to substantially lower AUC scores in all classifiers. This suggests that text-based generative models, although powerful in natural language contexts, may struggle to accurately capture the multivariate dependencies inherent to structured clinical tabular data. Interestingly, most models trained on SD showed improved performance on the external validation dataset (SLDB), which is not intuitively expected. This observation may be attributed to differences in sepsis onset characteristics, with patients in the SLDB dataset exhibiting an earlier onset of sepsis compared to those in MaDB. Therefore, the performance of the model increases when the appearance of sepsis occurs within a 24–48 h prediction window.

### 4.5. Experiment 4: Privacy Analysis

**Figure 5:**
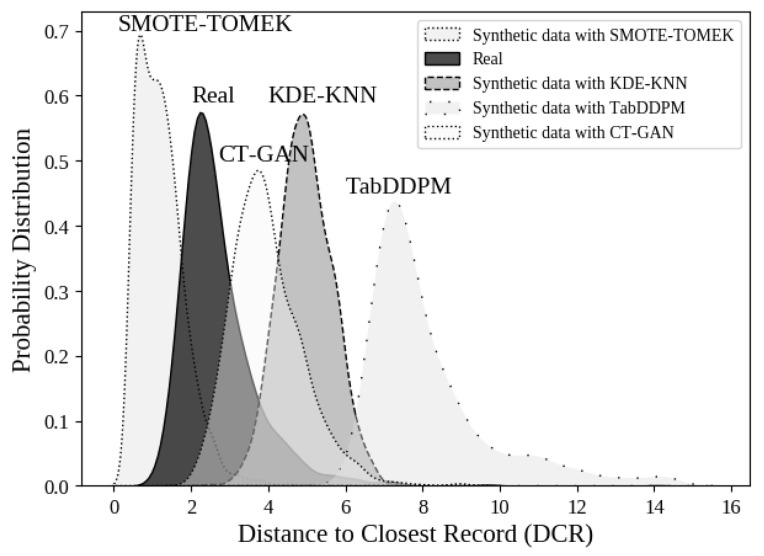
Probability distribution of the Distance to Closest Record (DCR) for real samples and synthetic samples generated with the 3 generation approaches evaluated in our experiments.

Furthermore, we evaluated the privacy-preserving characteristics of the best synthetic methods using the Distance to Closest Record (DCR) metric. The DCR is calculated as the Euclidean distance between a real sample and the closest synthetic sample. Low DCR values suggest that synthetic samples closely resemble real data points, which could compromise privacy requirements. In contrast, higher DCR values indicate that the generative model is capable of producing new records rather than replicating existing data. It is important to note that out-of-distribution data, such as random noise, can also yield high DCR values. Therefore, DCR must be evaluated alongside ML efficiency considerations [42]. Figure 5 presents the probability distributions of DCR for real samples ($d_{R-R}$) and the best generation approaches evaluated in previous experiments ($d_{R-S}$). The real data distribution is concentrated at low DCR values (approximately between 1 and 3), reflecting the expected proximity of real points to their nearest neighbors in the feature space. This distribution serves as a baseline for assessing the privacy-preserving properties of SD. A distribution shifted to the left of the real curve (lower DCR) indicates that the synthetic samples are very close to the real samples, suggesting lower privacy. In contrast, a distribution shifted to the right (higher DCR) indicates that synthetic samples are farther away from real data, implying a lower risk of re-identification. For SMOTE–TOMEK, the mean DCR value is 0.83, while for KDE-KNN, TabDDPM and CT-GAN, the values are 4.971, 7.463 and 3.9, respectively. Comparing these results with the mean distance between the real data, which is 2.715, we observe that TabDDPM, CT-GAN, and KDE-KNN demonstrate efficacy in generating SD that preserve privacy, exhibiting superior performance compared to SMOTE–TOMEK.

**Figure 6:**
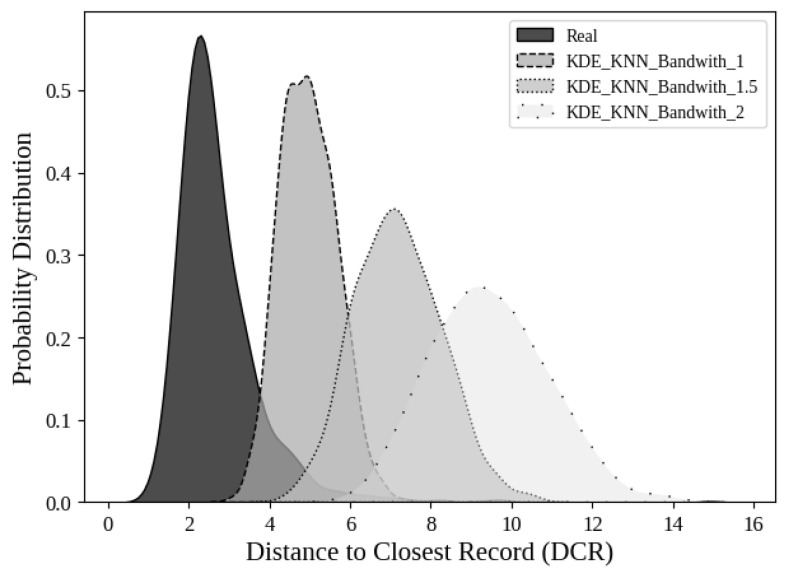
Probability distribution of the Distance to Closest Record (DCR) for real samples and synthetic samples generated with KDE-KNN adjusting the bandwith parameter.

A critical parameter in KDE that affects privacy is bandwidth. The bandwidth in KDE determines the width of the kernel function applied to each data point. A smaller bandwidth produces a narrow kernel, leading to a distribution that closely follows the original data, with sharp peaks and low smoothing. In contrast, a larger bandwidth spreads the influence of each point more broadly, resulting in a smoother and more generalized distribution. For this reason, higher bandwidth values are generated in synthetic samples that are more distant from the original data, thus enhancing privacy-preserving properties, as shown in Figure 6. However, this introduces a trade-off between privacy and utility, meaning that the bandwidth must be carefully chosen to ensure both sufficient distance between real and synthetic samples and a high level of realism.

Additionally, Appendix B provides a detailed analysis of the SD distributions. KDE-KNN and TabDDPM achieve the highest density and coverage scores, indicating a strong ability to approximate the real data distribution while preserving sample diversity. CT-GAN demonstrates moderate performance, whereas SMOTE–TOMEK exhibits lower coverage.

## 5. Discussion and Limitations

AI models have demonstrated significant potential to advance the biomedical field by improving patient outcomes, enabling personalized medicine, and facilitating early diagnosis. Developing these models requires datasets with enough data to capture the complexity of the diseases. However, in the biomedical domain, there is a lack of high-quality datasets, and patient data is under strong regulation. To address these challenges, SD generation has emerged as a promising solution. In healthcare, tabular data are a primary source of patient information, characterized by its inherent heterogeneity, including discrete and continuous features with diverse distributions. In this context, recent work has explored robust data-driven approaches for handling noisy medical datasets, such as outlier detection using iterative adaptive mini-minimum spanning tree generation, highlighting the importance of effective data quality control in biomedical applications [73]. For these reasons, it is paramount to carefully assess the synthetic samples generated in terms of quality and privacy concerns. In this study, we present an enhanced version of our method, KDE-KNN, which synergistically combines a model-driven approach KDE with a data-driven algorithm KNN to serve as a robust quality sample validator. This integrated framework not only ensures that the SD accurately reflects the underlying distribution of the original dataset but also incorporates a privacy validation mechanism using the DCR metric. The dual validation strategy is a key strength of our approach, as it has adjustable parameters to balance data quality and privacy according to specific application requirements. Moreover, KDE, a non-parametric statistical method, has consistently demonstrated its effectiveness in modeling small datasets. By combining KDE with KNN, our method improves the reliability of SD generation, making it particularly well-suited for biomedical applications where both data scarcity and stringent privacy regulations are common challenges.

When training ML models for sepsis detection using a balanced synthetic dataset, we observed improved performance on both the test set and the external validation cohort. In particular, the SVM classifier achieved the highest performance in the sepsis detection task. This improvement can be attributed to the correction of class imbalance, as the original real dataset was highly imbalanced, whereas the synthetic dataset provided a balanced representation of both classes. Although AUC was used as the primary evaluation metric in this study due to its threshold-independent nature, additional metrics such as sensitivity, specificity, and calibration are essential for clinical deployment. In this context, recent work conducted at Son Llàtzer Hospital has focused on clinically oriented sepsis detection systems evaluated on large-scale clinical data [74].

The comparative analysis of SD generation methods also provides relevant insights. KDE-KNN showed competitive performance compared to alternative approaches in terms of downstream predictive utility, while maintaining a favorable privacy profile. In contrast, the SD produced by the CHAT-GPT-based models (o1, o3-high, and 4o) exhibited poor utility for model training, leading to substantially lower AUC scores across all evaluated classifiers. The underperformance of LLM-based approaches may be explained by their original design objective, which is natural language modeling rather than the accurate generation of structured clinical tabular data. Unlike methods specifically designed for tabular synthesis, LLMs may not reliably capture complex multivariate dependencies, numerical precision, and class-conditional distributions, all of which are critical for preserving downstream predictive utility in biomedical applications.

In addition to utility, we evaluated the statistical similarity between real and synthetic data using distribution-based metrics such as the KS test. Low KS values indicate that the synthetic data closely match the real data distribution, reflecting high fidelity at the distribution level. However, this similarity may also suggest potential overfitting if synthetic samples replicate real instances too closely, which could increase the risk of privacy leakage. To mitigate this, we complement distribution-based metrics with distance-based measures such as the DCR, which explicitly capture sample-level similarity. By jointly analyzing both distributional and distance-based metrics, we provide a more comprehensive assessment of the trade-off between data fidelity and empirical disclosure risk. Lower DCR values indicate that synthetic samples lie very close to real data points, increasing the risk of re-identification or information leakage, while higher values suggest that synthetic samples are more distinct and therefore less likely to expose sensitive information. In particular, SMOTE–TOMEK produces a mean DCR value (0.83) that is significantly lower than the mean real-to-real distance (2.715). This indicates that synthetic samples generated by SMOTE–TOMEK are, on average, closer to real data points than real samples are to each other, suggesting a higher risk of privacy leakage. This behavior is expected, as SMOTE generates samples through interpolation between existing data points, which can lead to synthetic instances that closely resemble real individuals. In contrast, the evaluated generative approaches produce higher DCR values (4.971 for KDE-KNN, 3.9 for CT-GAN, and 7.463 for TabDDPM), indicating that these methods generate samples that are more distant from the original records.

Although the proposed approach incorporates a distance-based constraint to reduce similarity between real and synthetic samples, it is important to note that this mechanism provides an empirical notion of privacy rather than differential privacy [69]. In this context, the DCR metric is used as an empirical proxy for disclosure risk, capturing sample-level similarity between real and synthetic data. Future work will further extend this analysis by incorporating complementary privacy evaluation methods, including adversarial attacks and differential privacy-based approaches [75,76].

A limitation of our algorithm is scalability. Since each phenotype requires its own adjusted KDE, the approach is inherently best suited for scenarios involving a small number of phenotypes. Moreover, KDE itself presents challenges, such as convergence issues and sensitivity to bandwidth selection. Future research may explore more efficient parameter tuning strategies or approximate methods to overcome these limitations while maintaining data fidelity. Additionally, the results underscore the need for future investigations to include diverse diseases and datasets, due to the difficulty of obtaining compatible datasets for training and external validation purposes.

Finally, although this study formulates sepsis detection as a binary classification task using static features, future work will explore the extension of the proposed framework to time-series data, leveraging temporal modeling approaches such as recurrent neural networks or survival analysis methods to better capture the progression of sepsis over time.

### 5.1. Legal and Regulatory Framework Governing Synthetic Health Data

The development of AI models in the biomedical domain involves the processing of sensitive personal data, particularly health-related data, which are subject to strict legal safeguards. SD generation emerges as a promising strategy to reconcile the advancement of ML with the fundamental rights to privacy and data protection, especially in contexts governed by the European Union’s General Data Protection Regulation (GDPR) and the forthcoming AI Act. (a) GDPR and processing sensitive health data Under Regulation 2016/679 (GDPR), health data fall within the scope of special categories of personal data (Art. 4(15) and Art. 9 GDPR), the processing of which is, in principle, prohibited unless specific legal bases apply. Among these exceptions is the use of data for scientific research purposes, provided that appropriate safeguards are in place, including pseudonymization or anonymization techniques (Art. 9(2)(j) and Art. 89 GDPR).

SD, when properly generated and validated, can qualify as anonymous data that fall outside the scope of the GDPR. This interpretation is consistent with the Article 29 Working Party’s Opinion 05/2014 on Anonymization Techniques, which emphasized that once data are truly anonymous, they cease to be considered “personal data” under EU law. The European data-protection law sets a very high threshold for anonymization [77,78]. Whether that threshold can be met in practice depends on the deployment of rigorous privacy-enhancing techniques, including advanced statistical distance metrics and generative controls such as KDE-KNN.

### 5.2. The Emerging Regulation Landscape: The EU AI Act

The recently adopted AI Act (Regulation (EU) 2024/1689) by the European Union introduces a risk-based framework for AI applications. Healthcare-related systems, including diagnostic and decision-support tools, are classified as high-risk under this regulation. Compliance with the AI Act will require not only technical robustness, but also demonstrable respect for fundamental rights and data protection principles.

In this context, SD play a dual role: first, by reducing dependence on real personal data, it can lower the systemic risk of privacy breaches; and second, by enabling more equitable and reproducible datasets, it may help mitigate algorithmic bias [79,80,81] and improve model generalizability. The KDE-KNN method, as presented in this study, follows a privacy-aware design by incorporating statistical controls that regulate the similarity between real and synthetic samples (via the DCR metric), while preserving the utility of the synthetic dataset for training ML models.

Therefore, the use of SD generation techniques such as KDE-KNN aligns not only with technical excellence, but also with the evolving legal obligations imposed by the GDPR and AI Act. It reflects a legally sound and ethically defensible strategy for the deployment of AI in sensitive biomedical contexts, in line with the broader scholarly consensus that legal compliance must be integrated into the design and validation phases of AI development.

## 6. Conclusions

We proposed and evaluated KDE-KNN as a statistical method for generating synthetic tabular data. Without loss of generality, we assessed this method in terms of both utility and privacy protection through an extensive evaluation in the context of sepsis detection. Remarkably, when we trained ML models for sepsis detection with a balanced synthetic dataset, we obtained better results on both the test set and the external validation. In particular, an SVM classifier demonstrated the highest performance in the sepsis detection task, achieving 0.7128 in the test set and 0.7682 in external validation when trained with SD generated via KDE-KNN. In contrast, when trained on real data, its performance was 0.6194 and 0.6952, respectively. We attributed this improvement to the fact that the real dataset was highly imbalanced, while the synthetic dataset was balanced. Thus, KDE-KNN would also represent a promising approach for balancing data sets. In addition, our findings have been corroborated by validation in an external database, reinforcing the generalizability potential of our synthesis approach. Additionally, the results demonstrated that KDE-KNN reduces the risk of re-identification, with a mean DCR of 4.971 between synthetic and real data points, compared to 2.715 between real data points.

These findings indicate that KDE-KNN effectively captures the underlying population distributions of real data while generating high-quality synthetic samples that maintain statistical fidelity and controlled similarity to the original data. By balancing the trade-off between data utility and sample-level similarity, KDE-KNN produces representative synthetic datasets while reducing the likelihood of re-identification of individual records, making it a valuable tool for data-driven applications.

In our future work, we will extend our synthetic data generation methods to simultaneously consider multiple variables to create comprehensive digital phenotypes [82]. We will also consider synthetic signal generation, keeping in mind the privacy elements [83,84], and will combine privacy-preserving tabular and signal generation to generate comprehensive and realistic digital twins and avatars [85].

## Figures and Tables

**Table 1:** Description of the 27 variables included in the databases. Differences in distributions between cohorts were assessed using the non-parametric Mann–Whitney U test. *p*-values were adjusted for multiple comparisons using the Bonferroni correction.

Feature	Description	Adj. *p*-Value
F1_Heart_rate	Number of heartbeats per minute	<0.01
F2_Leukocytes	Cells of the immune system	<0.01
F3_Temperature	Body temperature	<0.01
F4_Respiratory_rate	Number of breaths a person takes per minute	<0.01
F5_Bilirubin	Compound originating from heme catabolism [54]	<0.01
F6_Blood_urea_nitrogen	Amount of urea nitrogen in the blood	<0.01
F7_Creatinine	The end product of creatine phosphate metabolism [55]	0.49
F8_Diastolic_bp	Blood pressure measurement	<0.01
F9_Fraction_of_inspired_o2	Fraction of oxygen present in the air that a person inhales	<0.01
F10_Systolic_bp	Blood pressure measurement	<0.01
F11_Thrombocytes	Blood cells	<0.01
F12_Lactate	Metabolite of glucose	<0.01
F13_Bicarbonate	Electrolyte [56]	<0.01
F14_C-reactive_protein	Molecule secreted in response to inflammatory cytokines [57]	<0.01
F15_Hemoglobin	Protein found in red blood cells	<0.01
F16_Lymphocytes	Cells of the immune system	<0.01
F17_Sodium	Electrolyte [56]	<0.01
F18_Pancreatic_lipase	Enzyme [58]	<0.01
F19_Procalcitonin	Peptide	<0.01
F20_Oxygen_saturation	Percentage of hemoglobin bound to oxygen [59]	<0.01
F21_Blood_glucose	Concentration of glucose	<0.01
F22_Chloride	Electrolyte [56]	<0.01
F23_Calcium	Electrolyte [56]	<0.01
F24_Potassium	Electrolyte [56]	<0.01
F25_Alanine_transaminase	Enzyme [60]	<0.01
F26_Aspartate_transaminase	Enzyme [60]	<0.01
F27_Age	Years	0.13

**Table 2:** Main characteristics of the databases, including the number of features and patients, as well as the mean, minimum and maximum time of sepsis onset (in hours) and the service where the data were collected.

DB	Patients	Features	Mean (t)	Min (t)	Max (t)	Hospital Service
MaDB	979 non-sepsis/296 sepsis	27	208.7	39.5	1385	ICU
SLDB	1014 non-sepsis/1014 sepsis	27	36	24	48	ICU/emergency

**Table 3:** Results of Experiment 1 using real data. The result is shown in terms of AUC ± variance as each model was trained and evaluated with 3 partitions.

Model	MaDB	SLDB
RF	** 0.6708±0.0169 **	0.6469±0.0313
SVM linear kernel	0.5426±0.0581	0.6120±0.0701
SVM rbf kernel	0.6194±0.0119	** 0.6952±0.0282 **
XGBoost	0.6547±0.0296	** 0.5753±0.0399 **
CatBoost	0.5571±0.0530	** 0.5657±0.0349 **

**Table 4:** Results of Experiment 2 integrating KDE and ML models. The result is shown in terms of AUC ± variance.

Data Generation Method	Trained Classifier	MaDB	SLDB
	RF	0.6820±0.0114	0.7450±0.0212
KDE-RF	SVM (linear)	** 0.6786±0.0067 **	0.7170±0.0069
	SVM (rbf)	0.6892±0.0087	0.7543±0.0095
	XGBoost	0.6863±0.0123	** 0.7368±0.0091 **
	CatBoost	0.6976±0.0114	** 0.6864±0.0091 **
	RF	0.6730±0.0165	0.6957±0.0563
KDE-SVM	SVM (linear)	0.6917±0.0053	0.7354±0.0128
	SVM (rbf)	0.6850±0.0114	** 0.7306±0.0137 **
	XGBoost	0.6814±0.0141	** 0.6478±0.0570 **
	CatBoost	0.6798±0.0281	** 0.6838±0.0270 **
	RF	0.6932±0.0074	0.7650±0.0049
KDE-KNN	SVM (linear)	0.7093±0.0063	0.7540±0.0040
	SVM (rbf)	0.7128±0.0062	** 0.7682±0.0016 **
	XGBoost	0.7029±0.0081	** 0.7421±0.0024 **
	CatBoost	0.7074±0.0105	** 0.6813±0.0210 **

**Table 5:** Results of Experiment 2, combining real and SD in the training set using the SVM model. The results are shown in terms of AUC ± variance.

% Real	% Synthetic	MaDB	SLDB
100	0	0.6194±0.0119	0.6952±0.0282
80	20	0.6828±0.0177	0.7329±0.0121
60	40	0.6874±0.0047	0.7319±0.0195
40	60	0.7033±0.0066	0.7515±0.0090
20	80	0.7160±0.0099	0.7589±0.0079
0	100	0.7129±0.0062	0.7682±0.0016

**Table 6:** Kolmogorov–Smirnov (KS) tests were conducted to assess whether one-dimensional synthetic distributions differ significantly from the corresponding real distributions.

Generation Method	Mean KS Test *p*-Value
SMOTE–TOMEK	0.1937
TabDDPM	0.2041
CHAT-GPT o1	0.3208
CHAT-GPT o3-high	0.3124
CHAT-GPT 4o	0.3179
CT-GAN	0.3480
KDE	0.2654
KDE-KNN	0.2924

## Data Availability

The data supporting this study’s findings are available upon reasonable request to the corresponding author. However, owing to privacy restrictions, the data are not publicly available. E.M.-F. (corresponding author) has full access to all study data and serves as the guarantor of the data integrity and accuracy of the data analysis.

## Abbreviations

The following abbreviations are used in this manuscript:

AI	Artificial Intelligence
AUC	Area Under the Curve
DCR	Distance to Closest Record
DMs	Diffusion Models
EHR	Electronic Health Records
GANs	Generative Adversarial Networks
ICU	Intensive Care Unit
KDE	Kernel Density Estimation
KNN	K-Nearest Neighbors
LLMs	Large Language Models
MaDB	Mannheim Database
ML	Machine Learning
rbf	Radial Basis Function
RF	Random Forest
SD	Synthetic Data
SLDB	Son Llàtzer Hospital Database
SMOTE	Synthetic Minority Oversampling Technique
SVM	Support Vector Machine
TPE	Tree-Structured Parzen Estimator

## Appendix A. Hyperparameter Grid Search

**Table A1:** Hyperparameter search space and optimal values for the SVM model.

Parameter	Type	Range/Options	Optimal Value
*C*	Integer	[1,10]	4
Kernel	Categorical	[linear,poly,rbf]	rbf
γ	Categorical	[scale,auto]	scale

**Table A2:** Hyperparameter search space and optimal values for the KNN model.

Parameter	Type	Range/Options	Optimal Value
n_neighbors	Integer	[1,30]	23
Weights	Categorical	[uniform]	uniform
Metric	Categorical	[manhattan,minkowski]	manhattan

**Table A3:** Hyperparameter search space and optimal values for the Random Forest model.

Parameter	Type	Range/Options	Optimal Value
n_estimators	Integer	[10,200]	120
max_depth	Integer	[2,32]	16
min_samples_split	Integer	[2,10]	4
min_samples_leaf	Integer	[1,10]	2

**Table A4:** Hyperparameter search space and optimal values for the CatBoost model.

Parameter	Type	Range/Options	Optimal Value
iterations	Integer	[100,150]	118
learning_rate	Float	[0.05,0.1]	0.0871
depth	Integer	[6,10]	9

**Table A5:** Hyperparameter search space and optimal values for the XGBoost model.

Parameter	Type	Range/Options	Optimal Value
n_estimators	Integer	[100,200]	148
max_depth	Integer	[4,10]	7
learning_rate	Float	[0.01,0.05]	0.0216
γ	Float	[0,0.3]	0.1664
subsample	Float	[0.5,0.8]	0.5931
colsample_bytree	Float	[0.5,0.9]	0.7361
scale_pos_weight	Float	[1.0,10.0]	8.3990

**Table A6:** Hyperparameter search space and optimal values for the TabDDPM model.

Parameter	Type	Range/Options	Optimal Value
d_layers	List	{[128,128],[256,256]}	[128, 128]
dropout	Float	[0.0,0.2]	0.0
num_timesteps	Integer	{1000}	1000
gaussian_loss_type	Categorical	{mse}	mse
scheduler	Categorical	{cosine}	cosine
steps	Integer	[20,000, 50,000]	20,000
lr	Float	$\left[1\times 10^{-3},\;2\times 10^{-3}\right]$	0.00196
weight_decay	Float	$\left\{0.0,\;1\times 10^{-5}\right\}$	0.0
batch_size	Integer	{256,512}	256

**Table A7:** Hyperparameter search space and optimal values for the CT-GAN model.

Parameter	Type	Range/Options	Optimal Value
epochs	Integer	[300,1000]	600
batch_size	Integer	{256,500}	500
generator_lr	Float	$\left[1\times 10^{-4},\;2\times 10^{-4}\right]$	2 $\times 10^{-4}$
discriminator_lr	Float	$\left[1\times 10^{-4},\;2\times 10^{-4}\right]$	2 $\times 10^{-4}$
generator_dim	List	{(128,128),(256,256)}	(256, 256)
discriminator_dim	List	{(128,128),(256,256)}	(256, 256)
pac	Integer	{5,10}	10
embedding_dim	Integer	{64,128,256}	128
generator_decay	Float	$\left\{0.0,\;1\times 10^{-6},\;1\times 10^{-5}\right\}$	1 $\times 10^{-6}$
discriminator_decay	Float	$\left\{0.0,\;1\times 10^{-6},\;1\times 10^{-5}\right\}$	1 $\times 10^{-6}$
log_frequency	Boolean	{True,False}	True

**Table A8:** Hyperparameter search space and optimal values for the SMOTE–Tomek method.

Parameter	Type	Range/Options	Optimal Value
sampling_strategy	Float	all	all
k_neighbors	Integer	{3,5,710}	7

**Table A9:** Large Language Models and generation setup used for synthetic patient creation.

Aspect	Description
Models used	{o1, o3-high, 4o}
Task	Synthetic clinical data generation (control vs sepsis)
Sample size	540 control + 540 sepsis patients
Number of variables	27 clinical features
Prompting strategy	Single instruction prompt (no iterative refinement)
Full prompt	You are an expert in bioinformatics and medicine. Can you generate 540 control patients and 540 sepsis patients with the following 27 variables: [Heart Rate (bpm), Leukocytes (cells/mcL), Temperature (Celsius), Respiratory Rate (breaths/min), Total Bilirubin (mg/dL), Blood Urea (mg/dL), Blood Creatinine (mg/dL), Diastolic Blood Pressure (mmHg), FiO2 (%), Systolic Blood Pressure (mmHg), Platelets (cells/mcL), Lactate (mmol/L), Bicarbonate (mmol/L), C Reactive Protein (mg/L), Hemoglobin (g/dL), Lymphocytes (cells/mcL), Sodium (mmol/L), Lipase (U/L), Procalcitonin (ng/mL), O2 Saturation (%), Blood Glucose (mg/dL), Chloride (mmol/L), Total Calcium (mg/dL), Potassium (mmol/L), GPT ALT (U/L), GOT AST (U/L), Age (years)].

## Appendix B. Experimental Results

**Table A10:** Results of the experiment using SD, reported as AUC ± variance across three independently generated synthetic datasets. Each ML classifier was trained on synthetic samples derived from a subset of the MaDB dataset.

Data Generation Method	ML Classifier	MaDB	SLDB
	RF	0.6651±0.0184	0.5480±0.0128
SMOTE–TOMEK	SVM (linear)	0.6363±0.0292	0.6984±0.0134
	SVM (rbf)	0.6771±0.0212	0.5437±0.0596
	XGBoost	0.6512±0.0013	0.5404±0.0242
	CatBoost	0.6521±0.0363	0.5865±0.0254
	RF	0.6942±0.0102	0.5187±0.0804
TabDDPM [42]	SVM (linear)	0.6697±0.0207	0.6446±0.046
	SVM (rbf)	0.7020±0.0095	0.6949±0.0246
	XGBoost	0.6861±0.0196	0.5070±0.0727
	CatBoost	0.6563±0.0325	0.5238±0.0608
	RF	0.5988±0.0045	0.6649±0.0036
CHAT-GPT o1	SVM (linear)	0.5485±0.0034	0.5686±0.0058
	SVM (rbf)	0.5603±0.0053	0.6048±0.0038
	XGBoost	0.5388±0.0035	0.5780±0.0167
	CatBoost	0.5184±0.0103	0.5828±0.0143
	RF	0.5611±0.0045	0.6263±0.0036
CHAT-GPT o3-high	SVM (linear)	0.5763±0.0064	0.6093±0.0066
	SVM (rbf)	0.5815±0.0022	0.6188±0.0015
	XGBoost	0.5477±0.0088	0.5794±0.0015
	CatBoost	0.5751±0.0115	0.5824±0.0092
	RF	0.5952±0.0038	0.6129±0.0011
CHAT-GPT 4o	SVM (linear)	0.5419±0.0022	0.5933±0.0021
	SVM (rbf)	0.5621±0.0043	0.5873±0.0035
	XGBoost	0.5735±0.0043	0.5675±0.0052
	CatBoost	0.5583±0.0118	0.5896±0.0148
	RF	0.5895±0.0351	0.5349±0.0693
CT-GAN	SVM (linear)	0.6247±0.0266	0.6048±0.0192
	SVM (rbf)	0.5811±0.0278	0.5284±0.0971
	XGBoost	0.5025±0.0316	0.5285±0.0518
	CatBoost	0.5352±0.0262	0.5140±0.0462
	RF	0.6495±0.0051	0.6261±0.00255
KDE	SVM (linear)	0.6449±0.0017	0.7202±0.0215
	SVM (rbf)	0.6748±0.0072	0.7114±0.0019
	XGBoost	0.6860±0.0229	0.5931±0.0138
	CatBoost	0.5859±0.0148	0.5854±0.0112
	RF	0.6932±0.0074	0.7650±0.0049
KDE-KNN [ours]	SVM (linear)	0.7093±0.0063	0.7540±0.0040
	SVM (rbf)	** 0.7128±0.0062 **	** 0.7682±0.0016 **
	XGBoost	0.7029±0.0081	0.7421±0.0024
	CatBoost	0.7074±0.0105	0.6813±0.0210

**Table A11:** Evaluation of SD generation methods using DCR for privacy, and coverage and density metrics for diversity and fidelity, respectively.

Generation Method	DCR	Coverage	Density
SMOTE–TOMEK	0.82	0.70	0.96
TabDDPM	7.46	0.94	0.82
CT-GAN	3.98	0.84	0.67
KDE-KNN	4.97	0.90	0.79
